# Temporising external fixation reduces loss of reduction compared with plaster splinting in ankle fracture-dislocations: a systematic review and meta-analysis of cohort studies

**DOI:** 10.1007/s00402-026-06413-1

**Published:** 2026-07-28

**Authors:** Vishwa Suravaram, Linda Solomon, Saiuj Bhat, Matthias Wittauer

**Affiliations:** 1 School of Medicine, University of Western Australia, Perth, Australia; 2 School of Medicine, Curtin University, Perth, Australia; 3 University of Basel, Basel, Switzerland

**Keywords:** Ankle fracture, Dislocation, Splint, External fixation, Complications, Trauma

## Abstract

**Purpose:**

The optimal temporising strategy for ankle fracture-dislocations is a highly debated topic. These unstable injuries are commonly managed with staged fixation to allow for soft tissue recovery, using either plaster splinting or ankle-spanning external fixation. This study aimed to compare the efficacy and safety of these two approaches.

**Methods:**

A systematic search of MEDLINE, Embase, Scopus, Cochrane CENTRAL, and Google Scholar was conducted. Comparative studies evaluating temporary external fixation versus plaster splinting in skeletally mature patients with ankle fracture-dislocations were included. Meta-analyses were performed to assess differences in loss of reduction prior to definitive fixation, soft tissue complications, and time to definitive fixation. Risk of bias was assessed using ROBINS-I tool, and certainty of evidence was appraised using GRADE.

**Results:**

Nine retrospective cohort studies comprising 1638 patients were included (589 external fixation, 1049 splinting). Loss of reduction occurred in 3% of injuries in the external fixation cohort compared with 14.6% in those managed with plaster splinting (RR 0.22; 95% CI 0.09–0.52). Rates of overall soft-tissue complications were similar between cohorts. The data regarding time to definitive fixation were highly heterogeneous across studies. Overall certainty of evidence was moderate for LOR and very low for Soft Tissue Complications and Time to ORIF.

**Conclusion:**

Temporary external fixation significantly reduces loss of reduction compared with plaster splinting in AFDs, without an increase in soft-tissue complications. External fixation may be advantageous in higher-risk fracture patterns or when delayed definitive fixation is anticipated. Further prospective studies are required to refine patient selection and optimise temporising protocols.

**Supplementary Information:**

The online version contains supplementary material available at 10.1007/s00402-026-06413-1.

## Introduction

Ankle fractures are among the most common injuries encountered in orthopaedic trauma, and constitute the third most common cause of fracture-related hospitalisations after hip and humeral fractures [1]. Within this spectrum of injuries, ankle fracture-dislocations (AFDs) represent a severe subset, characterized by unstable fracture patterns and soft tissue compromise [2]. These injuries place substantial tension on the skin and neurovascular structures, increasing the risk of neurovascular compromise, compartment syndrome, and skin necrosis [2]. Although some guidelines recommend early definitive fixation for unstable ankle fractures [3], operating on markedly oedematous tissues increases the risk of wound complications, including dehiscence and infection, with reported rates of soft tissue complications as high as 13% [4].

Consequently, clinical practice often favours a staged approach, involving fracture reduction and temporary stabilisation until soft tissues recover [3], typically achieved with plaster splinting or external fixation (EF). While plaster splinting is resource-efficient, external fixation is often preferred for AFDs [2, 5], as splint effectiveness relies on soft-tissue conformity rather than bony fixation [5, 6]. As swelling subsides, the splint may lose its ability to maintain alignment, with secondary loss of reduction (LOR) reported in 17–43% of cases [7, 8]. In addition, circumferential skin contact and pressure areas over bony prominences may delay soft-tissue recovery and predispose patients to blisters and skin necrosis [9]. Ankle spanning external fixation provides a more rigid and reliable maintenance of fracture reduction without interfering with the fracture zone. It is less likely to impede recovery of compromised skin and allows for regular neurovascular and soft-tissue assessment [7]. However, it carries a greater resource burden and introduces the risks of pin-site related complications like infection and iatrogenic fractures [10].

The choice between these temporising strategies remains debated and is often influenced by local resource constraints and fracture severity. Given the widespread nature of these injuries in everyday orthopaedic trauma practice, the aim of this systematic review is to synthesize the current evidence comparing plaster splinting and external fixation for initial temporising management of AFDs, assessing outcomes related to loss of reduction (LOR), soft tissue complications – inclusive of skin necrosis, pin site infections & surgical site infections, and time to definitive surgical fixation.

## Methods

### Reporting guidelines

All relevant aspects of the Cochrane Handbook for Interventional Systematic Reviews were followed, and the study was conducted according to the Preferred Reporting Items for Systematic Reviews and Meta-Analyses (PRISMA) guidelines [11, 12]. The protocol was registered on the International Prospective Register of Systematic Reviews (PROSPERO ID: CRD420261285814).

### Search strategy

A systematic literature search was conducted in Medline, Embase, Scopus, Cochrane CENTRAL, and Google Scholar databases from inception to 1 July 2025 with no language restrictions using the search strategy outlined in the Supplementary Material (Supplementary Table S1: Search Strategy). Database-specific strategies combined controlled vocabulary and keywords related to ankle fracture-dislocation, temporising management, external fixation, and plaster/splinting. Reference lists of included studies and relevant reviews were hand-searched to identify additional citations. Grey literature sources and trial registries were screened as feasible.

### Eligibility criteria and study selection

Study selection, data extraction, and risk of bias assessment were independently performed by two reviewers (VS and LS). Any discrepancies were resolved by consensus or, if necessary, by consultation with a third reviewer (SB).

Eligible studies were randomized or nonrandomized comparative studies and prospective or retrospective cohorts comparing temporary ankle-spanning external fixation with plaster or splint immobilization as initial temporising management for radiographically confirmed closed or open ankle fracture–dislocations in skeletally mature patients prior to definitive fixation. Studies of isolated dislocation, ankle fractures without dislocation, case series without a comparator, reviews, editorials, and conference abstracts were excluded.

### Outcomes

The primary outcome was LOR before definitive fixation to assess the efficacy of both temporising strategies. Several cohorts explicitly defined LOR as ≥ 5 mm of talar subluxation or joint incongruity, and the overwhelming majority of captured events necessitated a direct change in clinical management. Secondary outcomes included overall soft tissue complications and time to definitive fixation. Overall soft tissue complications included necrosis, surgical site infections (SSI), wound dehiscence, and pin-site infection.

### Data extraction

Data were extracted using pre-piloted forms. Extracted information included study characteristics (first author, year of publication, country, study design, sample size), participant demographics (mean or median age, sex distribution, BMI), fracture characteristics (open/closed, posterior malleolar fragment size, uni-/bi-/tri-malleolar) and outcome data. When multiple datasets overlapped, the most recent or comprehensive cohort was included. When necessary, corresponding authors of included studies were contacted for missing or unclear data. Additional data were received from Penning et al. [13].

### Quality assessment

Randomized controlled trials were evaluated using the Cochrane Risk of Bias 2 (RoB-2) tool [14] while observational studies were assessed using the ROBINS-I tool [15]. Cochrane’s GRADE framework was used to guide the overall certainty of evidence [16].

### Data analysis

Quantitative outcomes were pooled via meta-analysis using a random effects model with continuity correction for studies with no events. Sensitivity analyses were performed for each outcome by applying different models for the meta-analyses, including a random effects model with no continuity correction, Mantel-Haenszel model with or without continuity correction, and Peto model. Similar pooled estimates were achieved using all models for all outcomes. The I^2^ and Q statistics were used to assess the heterogeneity of included studies. All analyses were conducted in STATA version 16.

## Results

### Study and patient characteristics

**Fig. 1:**
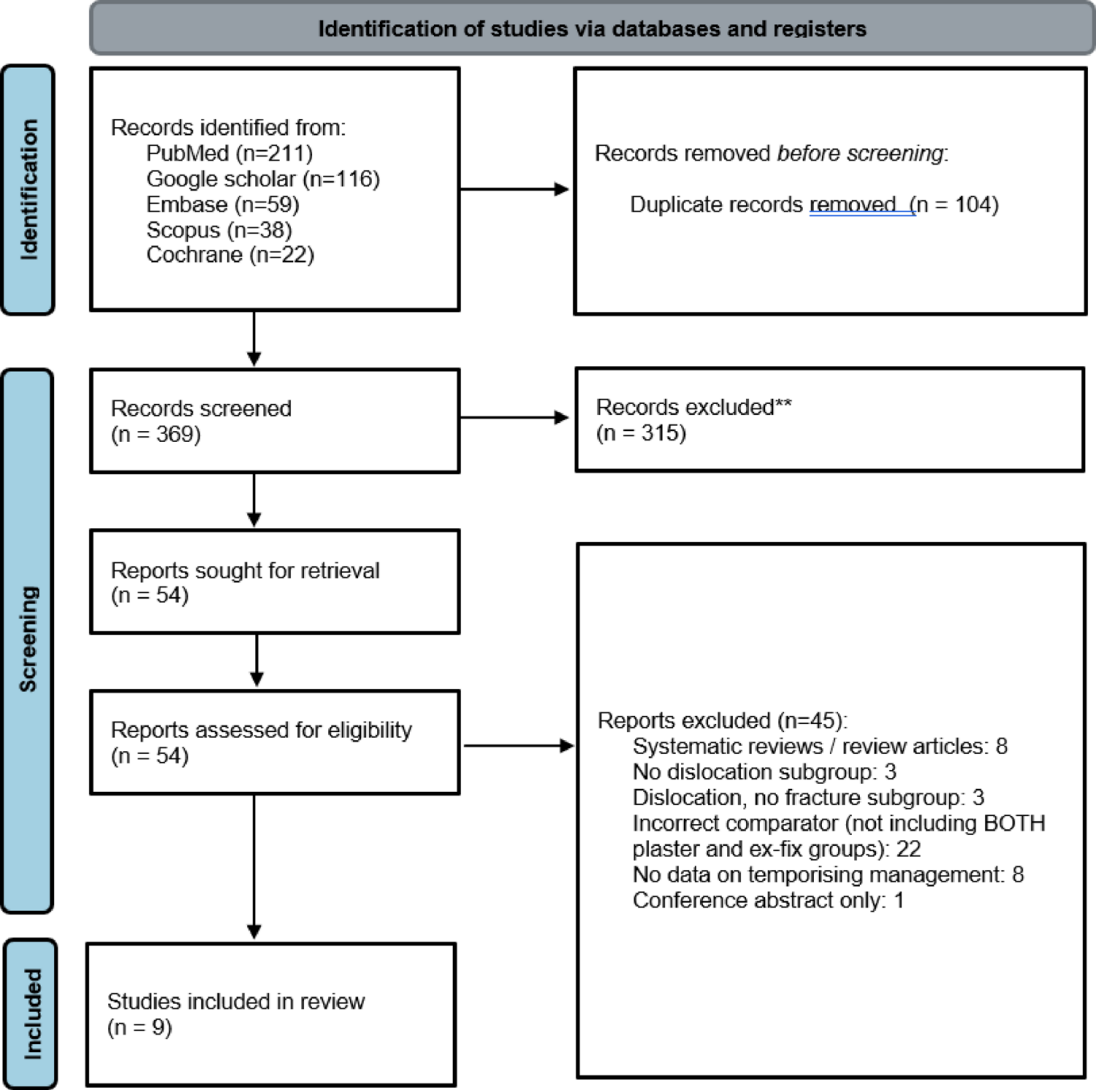
PRISMA flow diagram

Of 369 unique records, nine studies met the selection criteria and were included in the review [5, 7, 13, 17–22]. Figure 1 PRISMA Flow Diagram. All included studies were level III cohort studies published between 2020 and 2025. Table 1 summarises the main study characteristics and patient population for all studies included in the review. The total number of patients who underwent external fixation was 589, while 1049 had a plaster splint applied after fracture reduction. One study included open fractures. In this study, only Gustilo I and II fractures were splinted, whilst the external fixation cohort included Gustilo III fractures [13]. The primary outcomes specified by the individual studies were LOR [5, 7, 20, 22], soft-tissue complications [17–19], functional scores [21], or need for reoperation [13] while secondary outcomes included pin-site infection, surgical site infection after definitive fixation, and time to definitive fixation. Splinting protocols, criteria for acceptable reduction, and surveillance imaging schedules varied between centres and were seldom reported or detailed.

**Table 1:** Participant demographics

Study	Country	Primary outcome	Level of Eevidence	Sample size		Mean age (years)		Male (%)		BMI		Fracture characteristics	Reported outcomes
				EF	Splint	EF	Splint	EF	Splint	EF	Splint		
Theodorakis 2024	Italy	OMAS	III	21	31	52 (Range 32–75)	54 (Range 30–78)	38*	38*	NR	NR	Low energy trauma, Weber B and C, dislocations mostly posterior. Tscherne 0–1 soft tissues. Excluded open fractures	OMAS scores, complications, range of motion
Mandelka 2023	Germany	Loss of reduction	III	50	52	50 (SD 18)	44 (SD 15)	66	65	NR	NR	Exclusively uni-malleolar fractures. Excluded open fractures	LOR, soft tissue complications, time to ORIF
Penning 2024	Netherlands	Re-operaration for SSI	III	59	226	59 (31 IQR width)	50 (32 IQR width)	45.8	40.7	28 (8 IQR width)	27 (6 IQR width)	Mixed uni-,bi-,tri-malleolar fractures, high rate of Weber B and C, Included open fractures	LOR, Re-operation, SSI, implant removal
Gerlach 2022	Switzerland	Soft tissue complications	III	54	108	53 (Range 21–87)	50 (Range 16–83)	64	77	26 (Range 17–41)*	26 (Range 17–41)*	Bi- and tri- malleolar fractures, mostly Weber B, excluded open fractures	Time to surgery, Hospital stay, SSI, Skin necrosis
Buyukkuscu 2022	Turkey	Loss of reduction	III	48	69	48 (14 SD)	47 (12 SD)	63	52	NR	NR	Bi- or tri- malleolar fractures. Weber B or C.Excluded open fractures	LOR, skin necrosis, time to surgery, AOFAS, VAS
González-Morgado 2024	Spain	Soft tissue complications	III	56	138	63 (17 SD)	56 (18 SD)	NR	NR	NR	NR	Mixed cohort, predominantly bi- and tri- malleolar. Excluded open fractures.	LOR, complications, quality of reduction
Mandelka 2024	Germany	Loss of reduction	III	191	152	56.7 (95 CI 54.4–59.1)	52 (95 CI 49.2–54.8)	40.3	34.2	NR	NR	Bi- and tri-malleolar, excluded open fractures.	LOR, soft tissue complications, hospital stay, pin site infection
Joseph 2025	USA	Soft tissue complications	III	82	245	57 (IQR 42–67)^A^	57 (IQR 42–67)^A^	29	29.6	31.3 (26.8–36.6 IQR)*	31.3 (26.8–36.6 IQR)*	Tri-malleolar. Excluded open fractures	Soft tissue complications, BMI, demographics
Wawrose 2020	USA	Loss of reduction	III	28	28	46.8 (16.2SD)	57.2 (17.7 SD)	46	21	NR	NR	Trimalleolar, Weber B & C, excluded open fractures.	LOR, skin necrosis, time to surgery, complications

*AOFAS* American orthopaedic foot and ankle society score, *BMI* body mass index, *CRPS* complex regional pain syndrome, *EF* external fixation, *IQR* interquartile range, *LOR* loss of reduction, *NR* not reported, *OMAS* olerud-molander ankle score, *ORIF* open reduction and internal fixation (implied by reported outcomes), *PMF* posterior malleolar fragment, *SD* standard deviation, *SSI* surgical site infection, *VAS* visual analogue scale

^A^Demographics data includes non-dislocated ankle fractures

*Overall cohort data

### Loss of reduction (LOR)

**Fig. 2:**
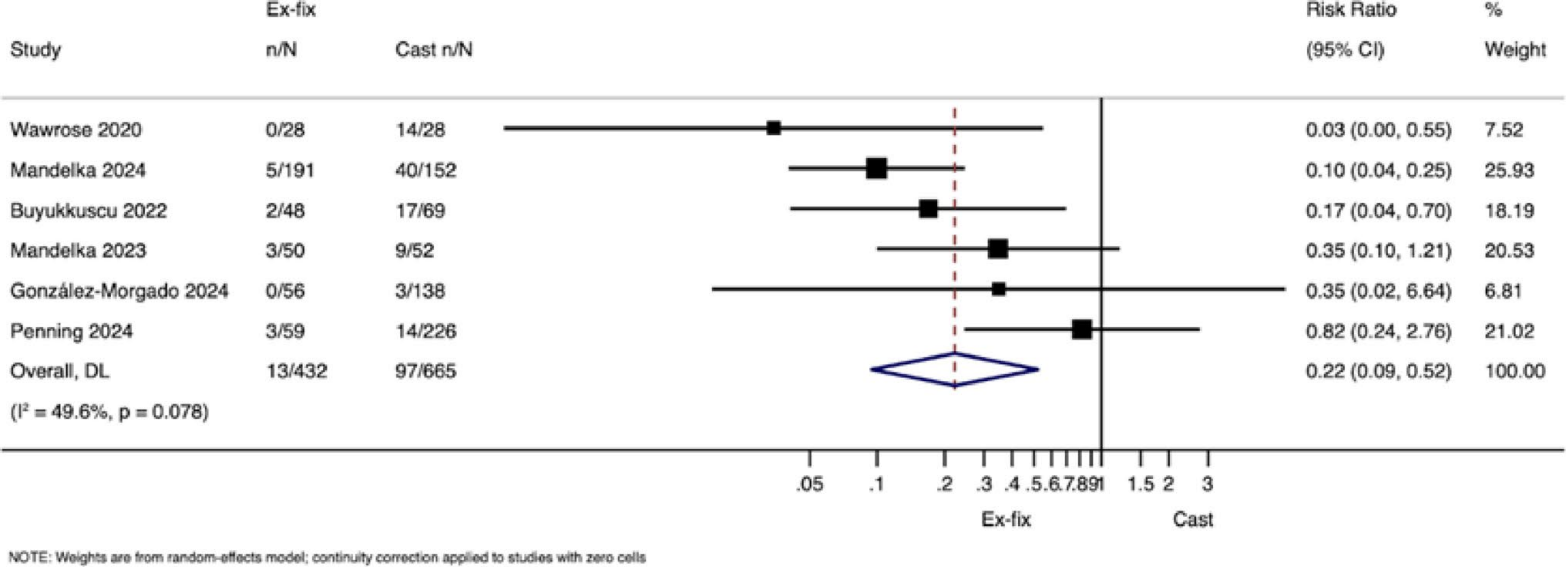
Loss of reduction forest plot

Six studies reported comparative data on LOR between external fixation and splinting [5, 7, 13, 18, 20, 22]. Across studies, LOR occurred in 3.0% (13/432) of ankles treated with external fixation compared with 14.6% (97/665) of those managed with plaster splinting. Pooled analysis demonstrated a consistently lower risk of LOR with external fixation (RR 0.22; 95% CI 0.09–0.52) with moderate heterogeneity (I2 = 49.6%). Figure 2 Loss of reduction forest plot. Several studies specifically evaluated risk factors associated with failure of the plaster splinting strategy as summarized in Table 2, with qualitative analysis identifying fracture morphology - Posterior Malleolar Fragment (PMF) size and Medial Clear Space as potential influencers of LOR.

**Table 2:** Study Characteristics & Outcomes

Study	LOR *n* (%)		Skin necrosis (*n* (%))		Overall soft tissue complications (*n* (%))		Pinsite infection (%)	Surgical site infections (*n* (%))		Time to definitive fixation (days)		Comments on predictors of LOR
	EF	Splint	EF	Splint	EF	Splint	EF	EF	Splint	EF	Splint	
Theodorakis 2024	-	-	-	-	2 (9.5)	5 (16.1)	-	-	-	4.1 +/- 1.95 SD	3.7+/−1.5 SD	Nil
Mandelka 2023	3 (6)	9 (17.3)	0 (0)	0 (0)	6 (12)	12 (23.1)	-	1 (2)	2 (3.8)	8.3 +/- 3.5 SD	8.7 +/- 3.7 SD	PMF size associated with LOR
Penning 2024	3 (5.1)	14 (6.2)	-	-	-	-	-	8 (13.6)	25 (11.1)	12 (9–14 IQR)	10 (8–13 IQR)	Nil
Gerlach 2022	-	25 (19.7)*	4 (7.4)	7 (6.5)	10 (18.5)	12 (11.1)	-	6 (11.1)	5 (4.6)	9.1 (4–21 Range)	6.4 (2–16 Range)	PMF size associated with LOR (*p* < 0.001). Identified threshold PMF ≥ 22.5% (Sens 61%, Spec 79%).
Buyukkuscu 2022	2 (4.2)	17 (24.6)	3 (6.3)	15 (21.7)	8 (16.7)	20 (29)	2 (4.2)	2 (4.2)	2 (2.9)	7 +/- 4 SD	11 +/- 5 SD	PMF size associated with LOR
González-Morgado 2024	0 (0)	3 (2.2)	-	-	5 (8.9)	15 (10.9)	0 (0)	5 (8.9)	11 (8)	15.1^A^	6^A^	Nil. However noted poor baseline characteristics in EF group for (*p* < 0.05): older age, Prevalence of PMF, blisters
Mandelka 2024	5 (2.6)	40 (26.3)	5 (2.6)	1 (0.7)	58 (30.4)	27 (17.8)	2 (1)	13 (6.8)	6 (3.9)	10.1 (5.7–14.5 95%CI)	10 (6–14 95% CI)	Lauge-hansen type / injury mechanism associated with LOR
Joseph 2025	-	-	-	-	6 (7.3)	24 (9.8)	-	-				Nil
Wawrose 2020	0 (0)	14 (50)	0 (0)	5 (17.9)	0 (0)	5 (17.9)	1 (3.6)	-		11.8^A^	27.8^A^	Nil
Overall	13 (3.0)	122 (15.8)	12 (3.2)	28 (6.85)	95 (17.9)	120 (14.6)	5 (1.5)	35 (7.6)	51 (6.84)			

*CI* confidence interval, *EF* external fixation, *IQR* interquartile range, *LOR* loss of reduction, *p* p-value, *PMF* posterior malleolar fragment, *SD* standard deviation, *Sens* sensitivity, *Spec* specificity, *SSI* surgical site infection

^A^Mean provided, no SD

-: Not applicable or not reported

*Overall cohort data

### Soft tissue complications

**Fig. 3:**
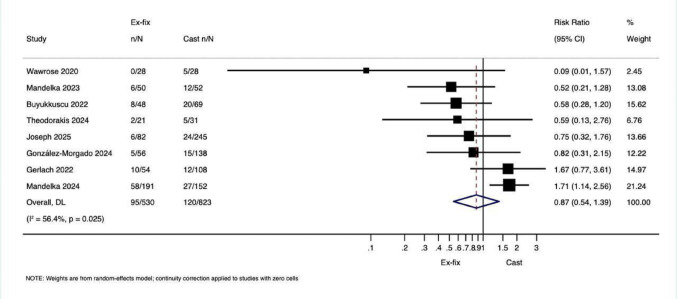
Overall soft tissue complications forest plot

Overall soft tissue complications were reported by eight studies [5, 7, 17–22]. Soft tissue complications occurred in 17.9% (95/530) in the external fixation group versus 14.6% (120/823) patients in the splinting group. No difference in risk was observed in the pooled analysis between the two groups (RR 0.87; 95% CI 0.54–1.39). Figure 3: Overall Soft Tissue Complications Forest plot.

Skin necrosis data were reported in five studies, with a prevalence of 3.2% (12/371) in the external fixation group versus 6.9% (28/409) in the plaster group. No difference in risk was observed between the two groups (RR 0.90; 95% CI, 0.23–3.47) [Supplementary Fig. 2: Skin necrosis forest plot] [5, 7, 17, 20, 22].

Rates of pin site infection were reported in four studies [5, 18, 20, 22], and represented 1.5% (5/323) of cases that underwent external fixation. There were no reported fixator removals in this group. No pin site related fractures were reported.

### Time to definitive fixation

**Fig. 4:**
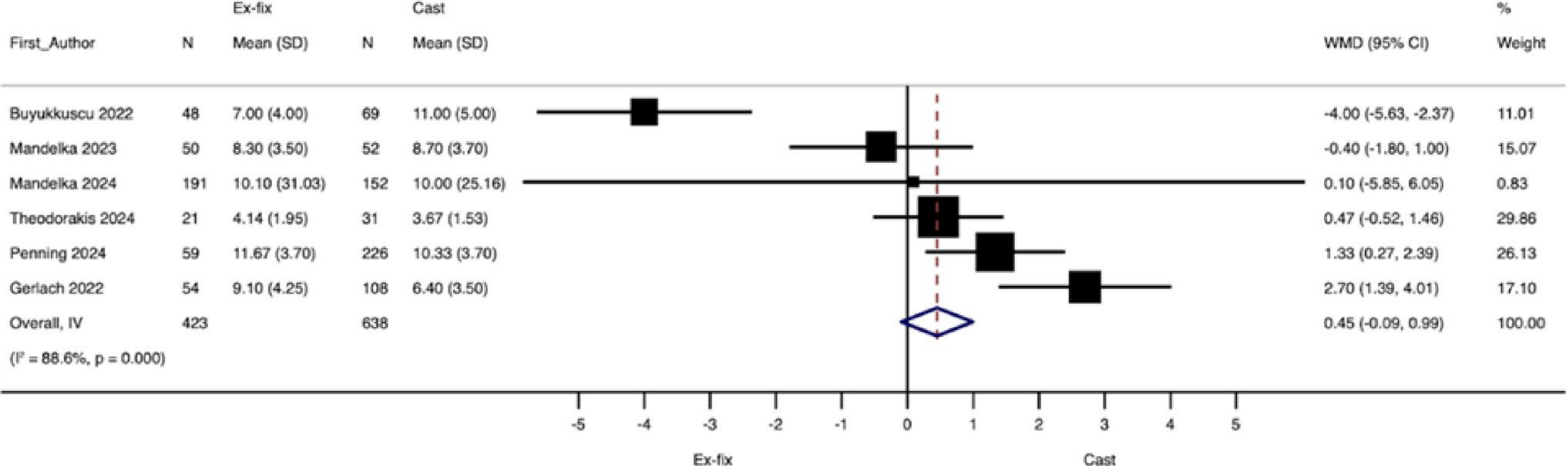
Time to ORIF forest plot

Across six studies reporting time to definitive fixation [5, 7, 13, 17, 20, 21], data were highly heterogeneous, with a pooled weighted mean difference of 0.45 days (95% CI − 0.09 to 0.99). Figure 4: Time to definitive fixation Forest plot.

### Quality assessment

Risk of bias was appraised using the ROBINS-I tool [Supplementary Fig. 1: ROBINS-I Risk of Bias]. Most studies exhibited moderate to serious risk of bias, driven primarily by confounding and participant selection, as more severe fracture-dislocations were frequently triaged to external fixation at surgeon discretion. Five studies achieved a moderate global risk rating [13, 17–19, 21], while the remainder were assigned serious risk [5, 7, 20, 22]. The certainty of evidence was assessed using the GRADE approach (Table 3). For loss of reduction, evidence was graded as Moderate certainty; there was overall serious risk of bias however the large magnitude of effect (RR 0.22) suggested a strong association and there was plausible residual confounding with confounding factors - injury severity, PMF, Soft tissue compromise, and presence of open fractures likely biasing against ex-fix. For secondary outcomes, certainty was graded as Very Low.

**Table 3:** Summary of evidence

External fixation compared to plaster splinting for temporising management of ankle fracture-dislocations					
People: Skeletally mature patients with ankle fracture-dislocations Settings: Emergency departments and trauma centres (USA, Germany, Switzerland, Spain, Netherlands, Turkey, Italy) Intervention: Temporizing ankle-spanning external fixation Comparison: Plaster splinting					

Outcomes	Absolute effect*		Relative effect (95% CI)	Number of studies	Certainty of the evidence (GRADE)
	Without external fixation	With external fixation			
Loss of reduction (prior to definitive fixation)	158 per 1000	35 per 1000	RR 0.22 (0.09 to 0.52)	6	⊕⊕⊕⊖ MODERATE ^1,2,3^
	Difference: 123 fewer per 1000 (95% CI 76 to 144 fewer)		I² = 49.6% *p* = 0.078		
Overall soft tissue complications (inclusive of pre- and post- ORIF)	146 per 1000	139 per 1000	RR 0.87 (0.54–1.39)	8	⊕⊖⊖⊖ VERY LOW ^4,5,6^
	Difference: 19 fewer per 1000 (95% CI 67 fewer to 57 more)		I² = 56.4 *p* = 0.025		
Time to definitive fixation (days to ORIF)	Range: 6.0–11.0 days	Range: 7.0–15.1 days	WMD 0.45 days (− 0.09 to 0.99)	6	⊕⊖⊖⊖ VERY LOW ^7,8,9^
	Difference: Mean 0.45 days longer with ExFix (95% CI 0.09 shorter to 0.99 longer)		I² = 88.6 *p* < 0.001		

*95% CI* 95% confidence interval, *RR* risk ratio, *WMD* weighted mean difference, *ExFix* external fixation, *ORIF* open reduction internal fixation

*The risk WITHOUT the intervention is based on the median control group risk across studies. The risk WITH the intervention (and its 95% CI) is based on the assumed risk in the comparison group and the relative effect of the intervention (and its 95% CI)

†GRADE Working Group grades of evidenceHigh = Very good indication of likely effect. Likelihood of substantial difference is lowModerate = Good indication of likely effect. Likelihood of substantial difference is moderateLow = Some indication of likely effect. Likelihood of substantial difference is highVery low = Does not provide reliable indication. Likelihood of substantial difference is very high

^1^ Risk of bias (LOR): Downgraded one level. ROBINS-I: Serious overall. Confounding by indication — more severe fracture-dislocations systematically triaged to ExFix. No propensity matching or adjustment for injury severity.

^2^ Large effect (LOR): Upgraded one level. RR 0.22 (78% RRR) meets threshold (RR < 0.5). Consistent across all six studies. Not upgraded two levels due to uncontrolled confounding.

^3^ Plausible residual confounding (LOR): Upgraded one level. Baseline characteristics (Table 1) indicate that factors associated with instability (open fracture, severe soft tissue compromise, larger PMF size) were disproportionately present in the ExFix cohort. Since these confounders would be expected to increase the rate of LOR in the ExFix group, the true intervention effect is likely underestimated by the observational data (GRADE 5.3.3)

^4^ Risk of bias (soft tissue): Downgraded one level. Serious risk of bias on ROBINS-I. Confounding by indication, variable definitions.

^5^ Inconsistency (soft tissue): Downgraded one level. I² = 56.4%, *p* = 0.025. Conflicting directions across studies. Variable outcome definition likely drive heterogeneity.

^6^ Imprecision (soft tissue): Downgraded one level. CI (0.54–1.39) crosses null, includes meaningful benefit and harm.

^7^ Risk of bias (time to ORIF): Downgraded one level. As above, plus confounding by institutional protocols, theatre availability, surgeon preference.

^8^ Inconsistency (time to ORIF): Downgraded two levels. Very serious. Conflicting directions across studies.

^9^ Imprecision (time to ORIF): Downgraded one level. CI (−0.09 to 0.99d) crosses null. Note the pooled estimate should be interpreted with caution given extreme heterogeneity.

## Discussion

The management of unstable ankle fracture-dislocations presents a significant challenge, centered on maintaining alignment while awaiting definitive fixation [2]. The aim of this manuscript was to compare the efficacy and safety of temporary external fixation versus splint immobilisation for ankle fracture-dislocations.

### Loss of reduction

External fixation was significantly more effective at preventing secondary LOR whilst awaiting definitive surgery. External fixation offers circumferential and adjustable control of length, alignment, and rotation, which may better resist talar displacement than plaster immobilisation [23]. In addition to providing provisional stability and soft tissue protection, the application of a spanning external fixator allows for CT imaging under traction. This improves visualization of articular impaction and fracture characteristics, thereby facilitating more accurate preoperative planning. However, suboptimal initial reduction with external fixation has been identified as a risk factor for revision surgery in unstable ankle injuries [6].

The relationship of the posterior malleolar fragment size and LOR may be clinically relevant. Across several studies, PMF size—rather than mere presence—was one of the key determinants of LOR in the temporising phase. Gerlach et al. (2022) established a critical threshold of 22.5% of articular surface involvement, above which the risk of LOR increased significantly [17]. This size-dependency was corroborated by Buyukkuscu et al. (2022) [5] and Mandelka et al. (2023) [7]. However, the evidence is not uniform. Mandelka (2024) found that only Lauge-Hansen type (Supination External Rotation & Pronation Abduction) was associated with LOR [20] - and recommended utilising Lauge-Hansen type alongside the presence of PMF when triaging to external fixation. In wider literature, Hecht et al. (2025), in a single arm study of patients managed with splinting, identified initial medial clear space widening (> 9 mm) as the strongest independent predictor of LOR [24].

The efficacy of splinting appears highly dependent on technique and clinical setting. Wawrose et al. reported high splint failure rates in non-specialist community settings [22], whereas González-Morgado et al. achieved a much lower LOR (2.2%) with close inpatient orthopaedic supervision [18]. When splinting fails, the consequences are substantial: conversion to external fixation ranged from 5.8 to 17.9%, entailing a second anaesthetic, renewed soft tissue trauma, and potential delay to definitive fixation. These rates likely underestimate true failure, as patients with immediate reduction failure were often excluded or converted before study enrolment [5, 7, 22].

### Soft tissue complications

Overall soft tissue complications, which included surgical site infections and skin necrosis, were comparable in the splinting and external fixation cohorts. Interpretation of the overall soft tissue outcomes was made difficult by the variable reporting standards and definitions of included complications, and aggregation of complications from both the temporising and postoperative phases. Where blistering was recorded in the primary data, it was captured as a preoperative baseline characteristic reflecting initial injury severity rather than as a postoperative complication of the temporising strategy itself. This variability in definitions and reporting likely drove the observed heterogeneity and may obscure the distinct risk profiles of each temporising method: splinting carries risk of ischemic pressure necrosis while external fixation carries risk of superficial bacterial entry. This is an important consideration in practice, as patients with skin necrosis face considerable delays in definitive fixation and may require additional coverage procedures [22]. Skin necrosis rates, in particular, were not different between the two strategies in our review. The low pin site infection rate observed in our analysis may reflect adherence to modern pin-care protocols, improved insertion techniques and the relatively short duration of frame application in this clinical context [25]. Among occurrences of pin site infections - there were no reported cases warranting pin removal. Our analysis also found no significant difference in postoperative surgical site infection rates between the two groups. This finding validates the staged protocol principles established by Sirkin et al. (1999) [26], confirming that the placement of transcutaneous pins does not “seed” the surgical field or increase deep infection risk, provided that pin sites remain healthy, are placed remote from planned incisions and any active pin infections are treated before conversion [19].

We acknowledge that a composite ‘overall soft tissue complication’ outcome has inherent limitations, and that reoperation for soft tissue complication represents a more clinically unambiguous endpoint. In the small subset of studies reporting this with sufficient granularity, event rates were low and no directional difference between strategies was apparent.

### Time to definitive fixation

Data on time to definitive fixation were highly heterogeneous, likely reflecting variations in institutional protocols rather than the efficacy of the temporising method. For example, Gerlach et al. [17] and González-Morgado et al. [18] reported significantly earlier definitive fixation in the splint group whilst Buyukkuscu et al. report the opposite [5]. The delays generally observed in the external fixation cohorts may be explained by best-practice rather than failure of the strategy. As demonstrated by Schepers et al., the critical factor for reducing wound complications is adhering to the “safe window” - either operating immediately (< 24 h) or delaying until the swelling / inflammatory phase resolves (> 7 days) [4]. The results of our study show that external fixation provides the surgeon with the necessary time to safely bridge the patient to the second window without losing fracture reduction.

### Strengths and limitations

To our knowledge, this is the largest meta-analysis to date comparing external fixation with splint immobilisation for AFDs, and utilises Cochrane best-practice for rigorous assessment of included studies with ROBINS-I and GRADE. Our analysis significantly updates the recent review by O’Connor et al. (2025) [27], which was limited by a search cutoff in October 2024. By incorporating three subsequent high-volume cohorts [13, 19, 21] —including the substantial data from Joseph et al. (*N* = 245) [19]—our study nearly doubles the total sample size (1,638 vs. 974), increasing power and allowing for greater precision in estimating complication risks.

Our findings must be interpreted in the context of the inherent selection bias in retrospective trauma studies. Formal assessment using ROBINS-I indicated an overall serious risk of bias, primarily driven by confounding by indication. Patients with open fracture, massive swelling, or severe fracture comminution were almost certainly triaged to external fixation.

Additional limitations include heterogeneity in plaster splinting techniques and acceptable reduction standards, as well as variable definitions of soft tissue complications and loss of reduction. The composite “overall soft tissue complication” rate reported in studies makes clinical interpretation more difficult, and we suggest future work utilise standardised criteria like the Centers for Disease Control and Prevention (CDC) Surgical Site Infection (SSI) criteria or reoperation-based soft tissue endpoints to provide more actionable guidance.

Moreover, studies were not uniformly designed to prospectively capture or screen for interim loss of reduction at predefined intervals, potentially leading to underestimation of event rates in some cohorts. Heterogeneity was moderate for the primary outcome and varied across the secondary endpoints. Reflecting these methodological constraints, the overall certainty of evidence using the GRADE approach was graded as very low for STC and Time to ORIF, and moderate for LOR.

In addition, a notable limitation of this study is the absence of cost analysis in the included literature. While external fixation was associated with lower rates of LOR, the economic implications of this strategy remain unclear. Several authors noted that external fixation entails increased costs through additional theatre time and prolonged hospital stays [13, 17, 19], while others observed that splint failure generates its own resource burden through repeat interventions and delayed definitive fixation [5]. Given the increasing emphasis on value-based care, future studies should incorporate cost analyses, including the financial impact of routine external fixation versus selective use based on risk of LOR, as well as the cost associated with repeat interventions. Such data are essential to inform evidence-based and economically sustainable treatment strategies.

## Conclusions

For ankle fracture-dislocations, temporising external fixation is associated with a lower rate of loss of reduction prior to definitive fixation compared to splinting, with comparable soft tissue complications and time to definitive fixation. Splinting nonetheless remains a reasonable, resource-efficient option for patterns that are stable once reduced and have an intact soft-tissue envelope, whereas external fixation is favoured for unstable reductions, high-risk patterns, already-compromised soft tissues, or anticipated delay to definitive fixation. In the absence of randomised data, this risk-stratified approach should be regarded as pragmatic guidance rather than a prescriptive protocol. To guide development of triage algorithms we suggest future research focus on prospective comparative studies that investigate the role of PMF, medial clear space, plaster technique as well as patient factors including comorbidities and compliance, in outcomes of external fixation vs. plaster splinting of ankle fracture dislocations.

## Supplementary Information

Below is the link to the electronic supplementary material.

Supplementary Material 1

Skin necrosis forest plot Supplementary Material 2

Risk of Bias Summary (The "robvis" traffic light plot) Supplementary Material 3

## Data Availability

The datasets generated during and/or analysed during the current study are included in the published article and its supplementary information files. Unprocessed data if required are available from the corresponding author on reasonable request.
