# Supplementary material for: Temporising external fixation reduces loss of reduction compared with plaster splinting in ankle fracture-dislocations: a systematic review and meta-analysis of cohort studies
Source: Arch Orthop Trauma Surg. 2026 Jul 28;146(1):271. doi: 10.1007/s00402-026-06413-1 (PMC13415331; doi:10.1007/s00402-026-06413-1)
Supplement: Supplementary file 1 — Supplementary Material 1 [file 402_2026_6413_MOESM1_ESM.docx]

Supplementary Table S1: Search Strategy

| **Database** | **Search Strategy Overview** | **Records Identified (n)** |
| --- | --- | --- |
| **PubMed** | Medical Subject Headings (MeSH) and keywords related to ankle fracture-dislocations, external fixation, and temporary immobilization (splinting or casting). | 211 |
| **Embase** | Combination of Embase Subject Headings (Emtree) and keywords for ankle fracture management, external fixators, and temporary immobilization. | 59 |
| **Scopus** | Keyword-based search strategy focused on the population (ankle fracture-dislocation) and interventions (external fixation vs. splinting). | 38 |
| **Cochrane Library** | MeSH descriptors and keyword search across "Ankle Fractures," "External Fixators," "Splints," and "Casts, Surgical". | 22 |
| **Google Scholar** | Targeted free-text search for clinical studies comparing external fixation versus splinting/temporary immobilisation in the context of ankle fracture-dislocations. (First 116 results were screened, results less relevant thereafter) | 116 |

Search reference export files available on request.
