# Supplementary figures and images for: Temporising external fixation reduces loss of reduction compared with plaster splinting in ankle fracture-dislocations: a systematic review and meta-analysis of cohort studies
Source: Arch Orthop Trauma Surg. 2026 Jul 28;146(1):271. doi: 10.1007/s00402-026-06413-1 (PMC13415331; doi:10.1007/s00402-026-06413-1)

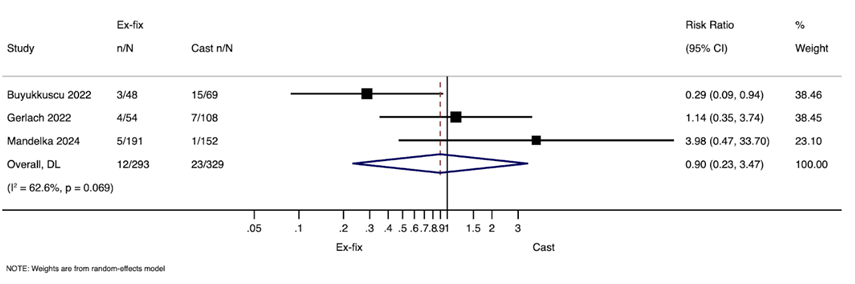

Supplement: Supplementary file 2 — Skin necrosis forest plot [file 402_2026_6413_MOESM2_ESM.png]

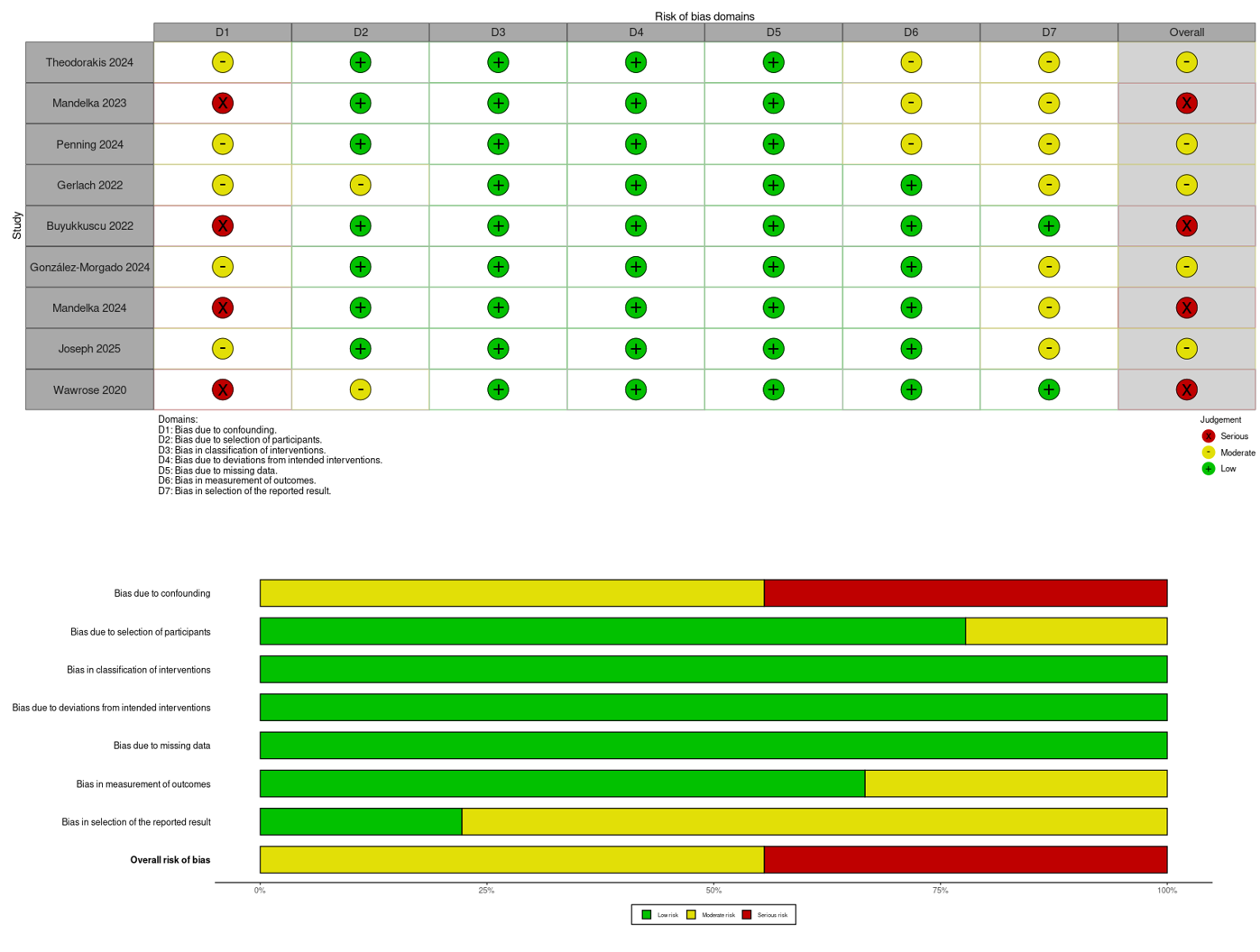

Supplement: Supplementary file 3 — Risk of Bias Summary (The "robvis" traffic light plot) [file 402_2026_6413_MOESM3_ESM.png]
